# Optimal Enteral Iron Dosing in Preterm and Very-Low-Birth-Weight Infants: A Systematic Review and Meta-Analysis

**DOI:** 10.7759/cureus.117030

**Published:** 2026-09-27

**Authors:** Anas Heissat, Dana A Al-Jarrah, Hashem T Kanaan, Aya N Alqurneh, Amro Alrashdan, Ahmed M Abu Mahmoud, Shahed Yacoub, Mina H Baaji, Mohammad M Almahasneh, Mihar Raouf, Elaf Ibdah, Abdullah H Haider

**Affiliations:** 1 Pediatrics, Jordanian Royal Medical Services, Amman, JOR; 2 Medicine and Surgery, Yarmouk University, ‏Amman, JOR; 3 Medicine and Surgery, University of Jordan, Amman, JOR; 4 General Practice, Jameel Al Totangi Hospital, Amman, JOR; 5 Medicine and Surgery, Mutah University, Amman, JOR; 6 Quality Control Microbiology, Retaj Pharmaceutical Industry, Mafraq, JOR; 7 Laboratory Medicine, The Hashemite University, Mafraq, JOR; 8 Medicine and Surgery, Balqaa University, Amman, JOR; 9 Medicine and Surgery, Marmara University, Istanbul, TUR

**Keywords:** anemia, enteral supplementation, iron deficiency, iron therapy, low birth weight, preterm infants

## Abstract

Preterm and very-low-birth-weight (VLBW) infants are particularly vulnerable to iron deficiency because of reduced fetal iron accretion, rapid postnatal growth, and phlebotomy losses, while excessive iron exposure may increase oxidative stress. The optimal enteral iron regimen therefore remains uncertain. We systematically reviewed randomized and non-randomized studies evaluating enteral iron dose, timing of initiation, formulation, or supplementation versus a comparator in preterm and eligible low-birth-weight infants. PubMed, Scopus, Web of Science, and supplementary sources were searched from inception through 21 June 2026. Two reviewers independently screened records, extracted data, and assessed risk of bias. Twenty-six reports representing 25 studies and 2,549 participants met the inclusion criteria. Quantitative synthesis was feasible for a limited subset of sufficiently comparable outcomes. Earlier versus later iron initiation was associated with higher hemoglobin in the primary random-effects analysis [mean difference (MD) 0.85 g/dL, 95% confidence interval (CI) 0.54 to 1.16; I² = 0%]; however, this finding was not robust in small-sample sensitivity analysis. No clear difference was observed for blood-transfusion requirement [risk ratio (RR) 0.38, 95% CI 0.07 to 2.17; I² = 69.5%] or reticulocyte count (MD −2.64 ×10⁹/L, 95% CI −30.97 to 25.69; I² = 80.3%). Given the limited quantitative evidence base, substantial uncertainty for several outcomes, and low-to-very-low certainty of evidence, firm conclusions regarding the optimal enteral iron regimen cannot be drawn. Further adequately powered comparative trials are needed to clarify the optimal dose and timing of supplementation.

## Introduction and background

Iron is indispensable to oxygen transport, mitochondrial electron transport, and myelination [1,2]. Fetal iron endowment largely accrues during the third trimester, so preterm infants begin life with reduced stores at the period of most rapid somatic and neural growth [3]. Preterm and very-low-birth-weight (VLBW) infants are therefore predisposed to iron deficiency through curtailed gestational accretion, rapid postnatal growth, and iatrogenic phlebotomy [3,4]. A substantial proportion develop iron deficiency or iron-deficiency anemia, with frequencies varying by birth weight, feeding practices, transfusion exposure, and diagnostic thresholds [4]. Conversely, sick infants often receive multiple red-cell transfusions, delivering a large iron load. Because preterm antioxidant systems are immature, this raises concern that non-transferrin-bound iron may catalyze reactive oxygen species formation and contribute to retinopathy of prematurity and bronchopulmonary dysplasia [2,5]. Enteral iron supplementation thus requires balancing the neurodevelopmental and hematological costs of deficiency against the oxidative risks of excess [6-8].

Current international guidance supports enteral iron supplementation in preterm and low-birth-weight infants. The World Health Organization (WHO) recommends enteral iron supplementation for human-milk-fed preterm or low-birth-weight infants who are not receiving iron from another source and suggests 2-4 mg/kg/day of elemental iron once enteral feeds are established. The European Society for Paediatric Gastroenterology, Hepatology and Nutrition (ESPGHAN) recommends 2-3 mg/kg/day beginning at approximately two weeks of age for VLBW infants, with supplementation individualized according to transfusion exposure and iron status [9,10].

However, these recommendations rest on a heterogeneous and often small evidence base. Trials have varied substantially in dose, timing, duration, iron preparation, co-interventions, population maturity, and outcome definitions. Previous syntheses concluded that enteral iron improves stores and reduces anemia, but effects on growth and neurodevelopment are uncertain, and higher doses confer little additional hematological benefit [4,11]. However, previous reviews were limited by substantial heterogeneity across intervention strategies and outcome definitions, relatively few direct comparisons of dose and initiation timing, and limited evidence concerning longer-term neurodevelopmental and safety outcomes. We therefore undertook this systematic review to synthesize the current evidence on enteral iron dose, timing, formulation, and supplementation strategies in preterm and eligible low-birth-weight infants.

## Review

Methods

Protocol and Registration

This systematic review was conducted in accordance with the PRISMA 2020 statement [12] and was prospectively registered in the Open Science Framework (OSF) [13].

Eligibility Criteria

Studies were eligible if they enrolled preterm infants (<37 weeks’ gestation), VLBW infants (<1500 g), or low-birth-weight infants satisfying the prespecified population criteria defined in the registered protocol; evaluated enteral iron supplementation compared with a different dose, initiation timing, formulation, placebo, or no supplementation; and reported hematological, iron-status, transfusion, growth, neurodevelopmental, oxidative-stress, or safety outcomes. Randomized, quasi-randomized, and comparative observational studies were eligible for comparative synthesis, while prespecified single-arm and before-after cohorts were retained for descriptive narrative synthesis. Reviews, editorials, conference abstracts without extractable data, animal studies, and studies outside the prespecified population were excluded. Studies in which iron dosing was not the operative exposure and term-only populations were excluded unless an eligible subgroup was reported separately. Companion publications originating from the same underlying cohort were linked and counted as a single study.

Information Sources and Search Strategy

PubMed, Scopus, and Web of Science were searched from inception through 21 June 2026. Supplementary searches were conducted in Google Scholar, the WHO International Clinical Trials Registry Platform (ICTRP), and the Open Science Framework (OSF). Search strategies were developed around four core concepts: preterm or very-low-birth-weight infants, enteral iron supplementation, iron dose or timing of initiation, and hematological or growth outcomes. Terms were combined using Boolean operators and adapted to the indexing and search syntax of each source. No date or language restrictions were applied. Reference lists of relevant systematic reviews and included studies were also screened for additional eligible records.

Study Selection and Data Extraction

Records were de-duplicated before title-and-abstract screening, followed by full-text assessment against the prespecified eligibility criteria. Two reviewers independently performed screening, with disagreements resolved by consensus. Two reviewers also independently extracted study design, country and setting, sample size, gestational-age and birth-weight criteria, feeding context, iron formulation, dose, initiation timing and duration, comparator, co-interventions, and reported outcomes into a piloted extraction workbook. Companion publications were linked to avoid double-counting participants. Extracted values and pooled estimates were cross-checked against the extraction workbook and statistical output files.

Risk of Bias

Randomized and quasi-randomized trials were assessed using the revised Cochrane risk-of-bias tool (RoB 2) [14]. Non-randomized comparative and before-after studies were assessed with Risk of Bias in Non-randomized Studies of Interventions (ROBINS-I) [15]; single-arm cohorts were retained for descriptive synthesis only. Domain-level and overall judgments were summarized from the extracted ratings.

Outcomes

Outcomes were organized into clinically meaningful domains: hemoglobin; ferritin and iron-status biomarkers [serum iron, transferrin saturation, soluble transferrin receptor (sTfR), reticulocyte indices, zinc protoporphyrin/heme ratio (ZnPP/H), reticulocyte hemoglobin content (RET-He)]; iron deficiency and iron-deficiency anemia; red-cell transfusion; growth; neurodevelopment; oxidative stress; neonatal morbidity; and exploratory microbiome outcomes.

Statistical Analysis

For each outcome with at least two studies reporting sufficiently compatible data, effect estimates were pooled using an inverse-variance random-effects model with the DerSimonian-Laird estimator [16]. Mean differences (MDs) were used for continuous outcomes and risk ratios (RRs) for dichotomous outcomes, with 95% confidence intervals (CIs). Statistical heterogeneity was quantified using I², τ², and Cochran’s Q. Because the number of studies contributing to individual meta-analyses was very small, robustness was examined by recomputing each pooled estimate using a restricted maximum-likelihood model with the Hartung-Knapp adjustment [17]. Studies with outcomes that could not be harmonized quantitatively, as well as single-arm cohorts, were synthesized narratively. Narrative findings were organized according to intervention comparison (dose, initiation timing, supplementation versus no supplementation, and formulation or route) and clinical outcome domain. Publication bias was planned to be assessed by funnel-plot inspection and Egger’s regression only when at least 10 studies contributed to an outcome. Certainty of evidence was evaluated using the grading of recommendations assessment, development and evaluation (GRADE) framework [18].

Results

Study Selection

The flow of records through identification, screening, eligibility, and inclusion is shown in Figure 1. A total of 720 records were identified, including 689 records from databases and 31 records from websites or grey literature. After removal of 26 duplicates and 69 records removed for other reasons during import and reconciliation, 625 records were screened by title and abstract. Of these, 568 were excluded, leaving 57 full-text reports sought and retrieved for eligibility assessment. Thirty-one full-text reports were excluded with reasons, leaving 26 reports of included studies, representing 25 unique studies that met the eligibility criteria. Overall, the included studies enrolled 2,549 participants. Fifteen studies were randomized or quasi-randomized trials, while ten were non-randomized or single-arm cohorts. Four reports, representing three independent trial families, contributed data to at least one quantitative meta-analysis; each pooled outcome included exactly two studies.

**Figure 1:**
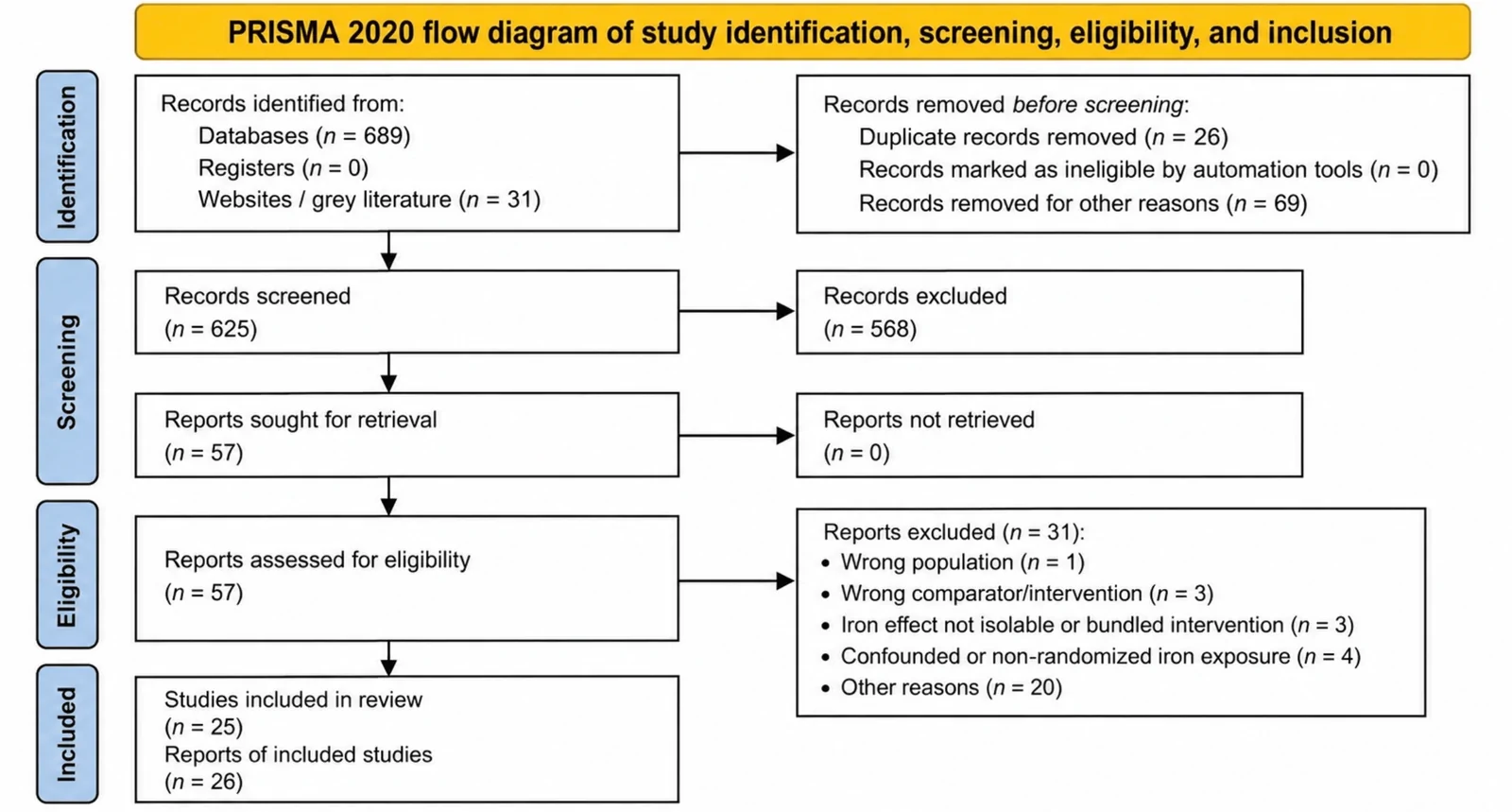
PRISMA 2020 flow diagram of study identification, screening, eligibility, and inclusion The 25 included studies were represented by 26 reports. Four reports, representing three independent trial families, contributed to at least one quantitative meta-analysis; each pooled outcome included two studies. The remaining evidence was synthesized narratively.

Characteristics of Included Evidence

The included studies were published between 1974 and 2024 and were conducted across Europe, North America, Asia, and the Middle East. Study designs ranged from double-blind randomized trials to prospective and retrospective cohorts. Enteral iron doses ranged from approximately 1 to 16 mg/kg/day, initiation varied from the first two postnatal weeks to two months of age, and follow-up ranged from several weeks to multiple years. Comparisons included higher versus lower dose, earlier versus later initiation, iron versus placebo or no supplementation, alternative formulations, and route comparisons; several trials included erythropoietin co-intervention (Table 1).

**Table 1:** Study design and intervention characteristics RCT: randomized controlled trial; QI: quality improvement.

Study	Design	N enrolled/recruited	Comparison
Nazir et al. [19]	Double-blind RCT	64	12 vs 6 mg/kg/day
Aggarwal et al. [20]	Double-blind RCT	73	3 mg/kg/day vs placebo
Berglund et al. [21]	Double-blind RCT	285	0 vs 1 vs 2 mg/kg/day
Kivivuori et al. [22]	Open RCT	45	Oral vs IM iron
Bader et al. [23]	RCT	30	16 vs 8 mg/kg/day
Arnon et al. [24]	RCT	116	2 wk vs 4 wk start
Jansson et al. [25]	RCT	31 enrolled; 28 analyzed	3 wk vs 2 mo start
Fujiu et al. [26]	RCT	24	4 vs 0 mg/kg/day
Franz et al. [27]	RCT	204	Early vs late initiation
Carnielli et al. [28]	RCT	63	EPO+IV vs EPO alone vs control
Barclay et al. [29]	RCT	62	13.8 vs 7 vs 0 mg/day
Naude et al. [30]	RCT	69	Ferrous sulfate vs nonionic FeIII
Friel et al. [31]	RCT	58	High vs normal formula iron
Arnon et al. [32]	RCT	68	2 wk vs 4 wk start
Joy et al. [33]	Single-blind RCT	104	2 wk vs 6 wk start
Rabe et al. [34]	Before-after QI	40	Multicomponent guideline
Lamport et al. [35]	Before-after cohort	100	Ferritin-guided vs routine
Olivares et al. [36]	Prospective cohort	84	Supplemented vs non-supplemented
Miller et al. [37]	Nonrandomised pilot	36	Iron-guided vs standard care
Li et al. [38]	Prospective cohort	63	By baseline iron status
Bahr et al. [39]	Retrospective QI	543	Single guideline cohort
Bagna et al. [40]	Retrospective cohort	300	Iron sulfate vs bisglycinate
Braekke et al. [41]	Single-arm	26 recruited; 21 analyzed	Single arm (pre/post)
Brozovic et al. [42]	Single-arm cohort	47	Single arm
VanOrmer et al. [43]	Single-arm pilot	14	Single arm (pre/post)

*Iron Dose Comparisons* 

Several trials compared higher with lower enteral iron doses. Nazir et al. [19] (12 vs 6 mg/kg/day during erythropoietin therapy) and Bader et al. [23] (16 vs 8 mg/kg/day) found that higher doses conferred no additional hematological benefit, with comparable ferritin and reticulocyte responses across arms. Barclay et al. [29] (13.8 vs 7 vs 0 mg/day) reported higher ferritin but lower erythrocyte superoxide dismutase activity in the highest-dose group. Berglund et al. [21] (0 vs 1 vs 2 mg/kg/day) found a lower prevalence of iron deficiency at higher doses.

Early Versus Later Initiation

Timing was addressed by Franz et al. [27], Joy et al. [33], Jansson et al. [25], Fujiu et al. [26], and Arnon et al. [24,32]. Earlier initiation was generally associated with improved iron stores and a reduced need for later transfusion; Joy et al. [33] reported higher ferritin, hemoglobin, and mean corpuscular hemoglobin concentration (MCHC) in the early group, and Franz et al. [27] reported fewer late transfusions with earlier iron. These studies provided compatible data for the quantitative analyses. Iron versus placebo or no supplementation. Fujiu et al. [26], Aggarwal et al. [20] (retained as a pre-specified subgroup in term low-birth-weight infants), and Olivares et al. [36] indicated improved hemoglobin or iron stores with supplementation.

Iron Formulation and Route Comparisons

Naude et al. [30] compared ferrous with nonionic ferric iron and found comparable efficacy with a non-significant trend toward better gastrointestinal tolerance for the ferric preparation. Bagna et al. [40] reported that iron bisglycinate chelate at 0.75 mg/kg/day achieved erythropoietic responses comparable to iron sulfate at 3 mg/kg/day. Kivivuori et al. [22] compared oral with intramuscular iron during erythropoietin therapy, with similar hematological responses by route. Transfusion outcomes were reported by Franz et al. [27], Carnielli et al. [28], Rabe et al. [34], and Lamport et al. [35], with earlier or protocolized approaches associated with fewer transfusions, though designs were heterogeneous and often confounded. Neurodevelopmental outcomes were reported by Li et al. [38], Friel et al. [31], and Franz/Steinmacher [27,44] with mixed findings based on adjusted analyses. Safety and oxidative-stress outcomes [29,41] were reported without consistent evidence of increased harm.

Risk of Bias

Risk of bias was assessed independently by two reviewers. The randomized or quasi-randomized trials (n=15) were appraised using RoB 2 [14], and the non-randomized comparative and before-after studies were assessed with ROBINS-I [15]; single-arm cohorts were retained for descriptive synthesis only. Across the randomized trials, most were rated as having some concerns, driven principally by unclear allocation concealment and open-label administration inherent to dose- and timing-comparison designs, with a minority at high risk owing to attrition or post-randomization exclusions. Non-randomized comparative studies were generally judged at serious risk under ROBINS-I, reflecting confounding by indication, historical-control comparisons, and time-varying co-interventions. RoB 2 domain-level judgments are shown in Figures 2, 3; ROBINS-I judgments are summarized narratively.

**Figure 2:**
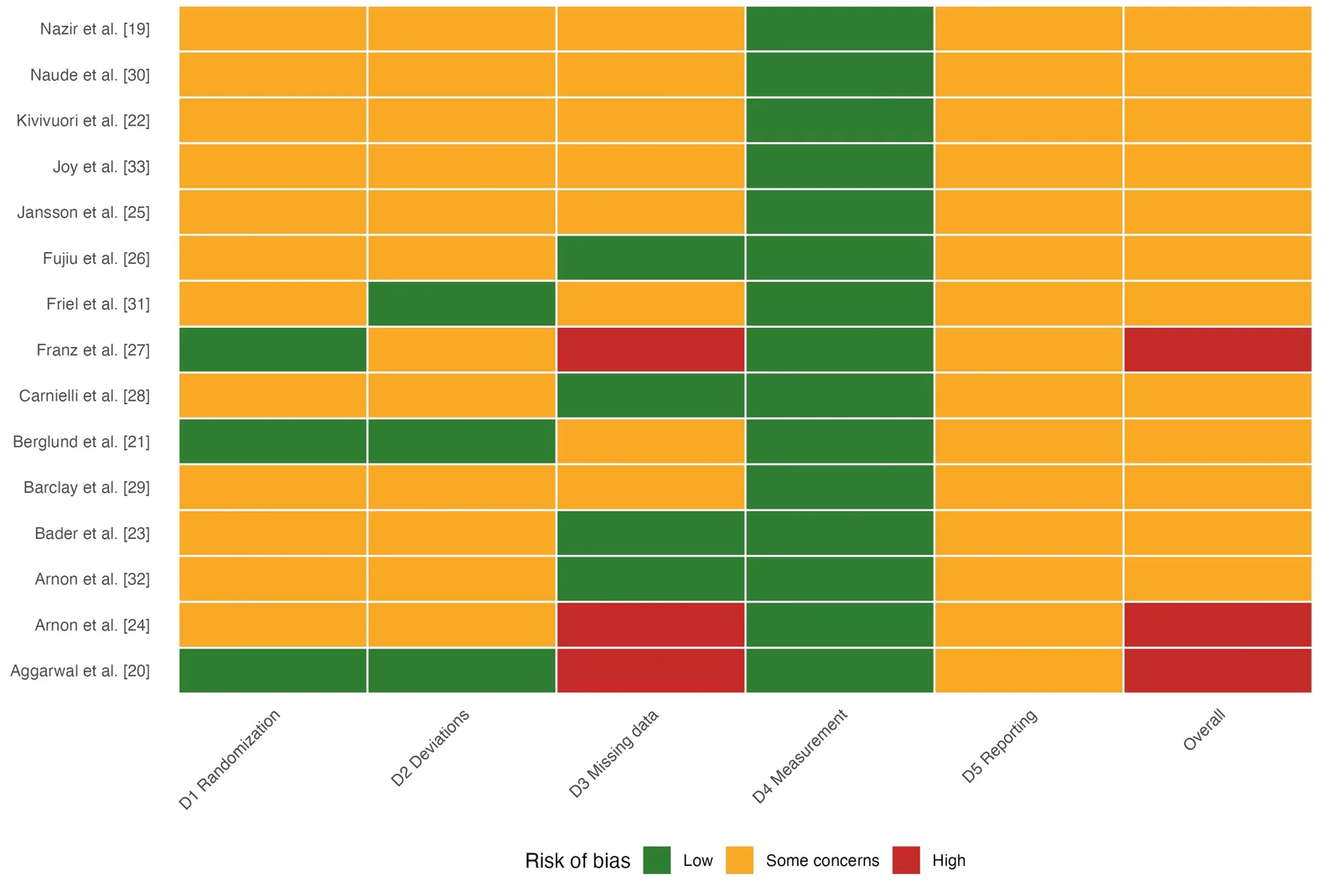
RoB 2 traffic-light plot for randomized and quasi-randomized trials of enteral iron strategies in preterm or eligible low-birth-weight infants RoB: risk-of-bias.

**Figure 3:**
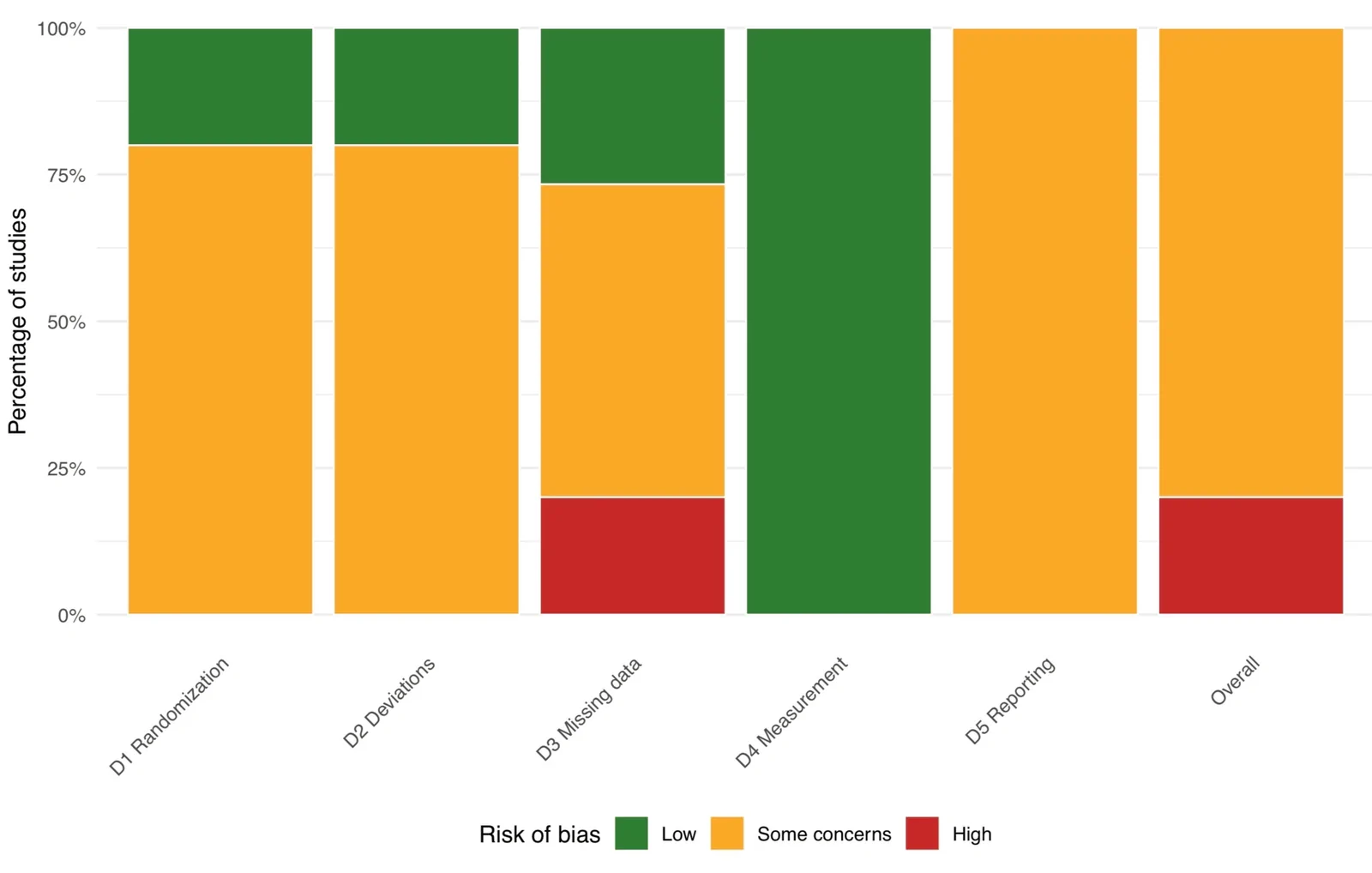
RoB 2 domain summary for randomized and quasi-randomized trials RoB: risk-of-bias.

Quantitative Synthesis

Overall, four study reports, representing three independent trial families, contributed to at least one quantitative meta-analysis. For hemoglobin, the DerSimonian-Laird random-effects model yielded a mean difference of 0.85 (95% CI 0.54 to 1.16; I² = 0%) favoring early initiation, but the Hartung-Knapp adjustment produced a confidence interval crossing the null (95% CI −0.60 to 2.29), indicating sensitivity to model choice. For blood transfusion, the pooled risk ratio was 0.38 (95% CI 0.07 to 2.17; I² = 69.5%) with substantial heterogeneity; the Hartung-Knapp interval was extremely wide and uninformative. For reticulocyte count, the mean difference was −2.64 (95% CI −30.97 to 25.69; I² = 80.3%), again with considerable heterogeneity and an extremely wide Hartung-Knapp interval (−186.31 to 181.02). Forest plots for these outcomes are shown in Figures 4-6, and the pooled estimates are summarized in Table 2.

**Figure 4:**
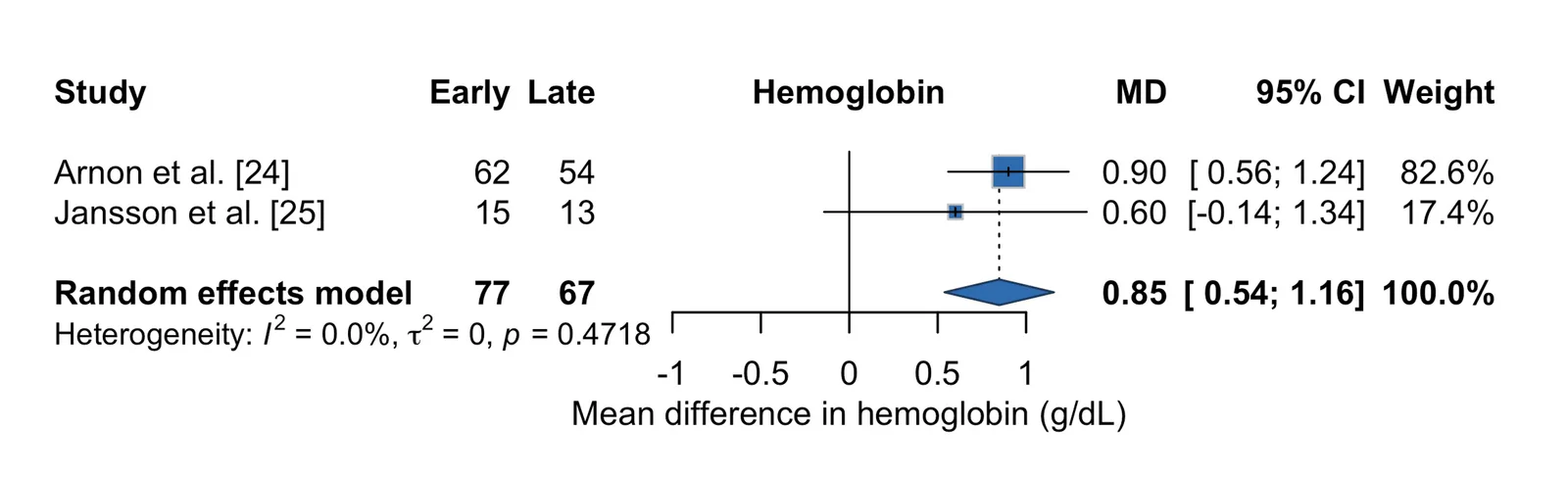
Hemoglobin mean difference by timing of iron initiation Forest plot of hemoglobin mean difference for early versus late enteral iron initiation. The pooled estimate uses an inverse-variance DerSimonian–Laird random-effects model. Positive values favor early initiation.

**Figure 5:**
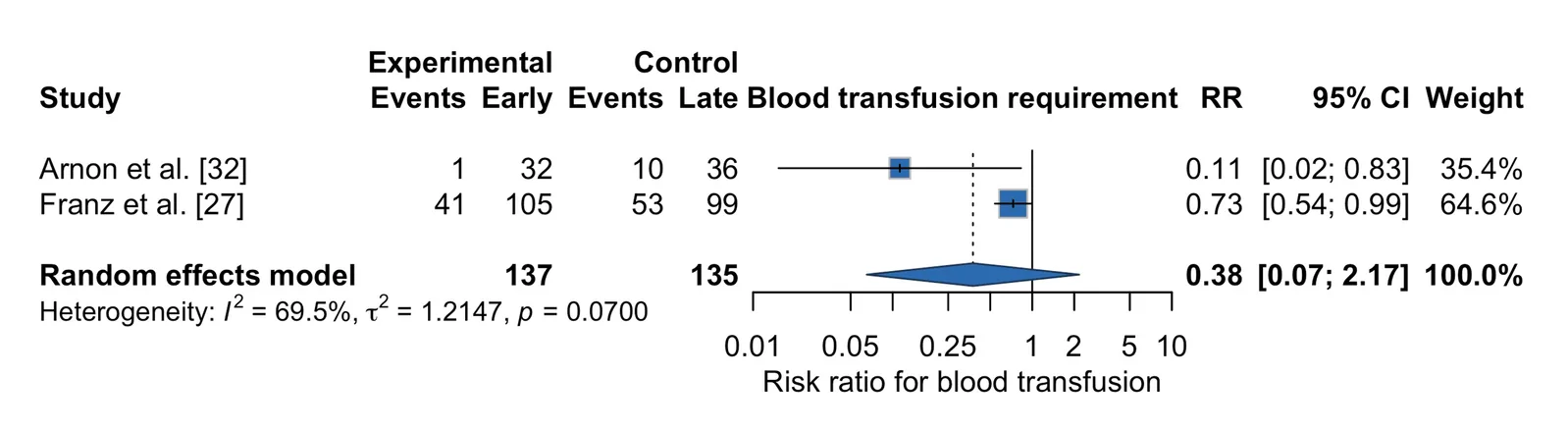
Blood-transfusion risk ratio by timing of iron initiation Forest plot of blood-transfusion risk ratio for early versus late enteral iron initiation. The pooled estimate uses an inverse-variance DerSimonian–Laird random-effects model. Risk ratios below 1 favor early initiation.

**Figure 6:**
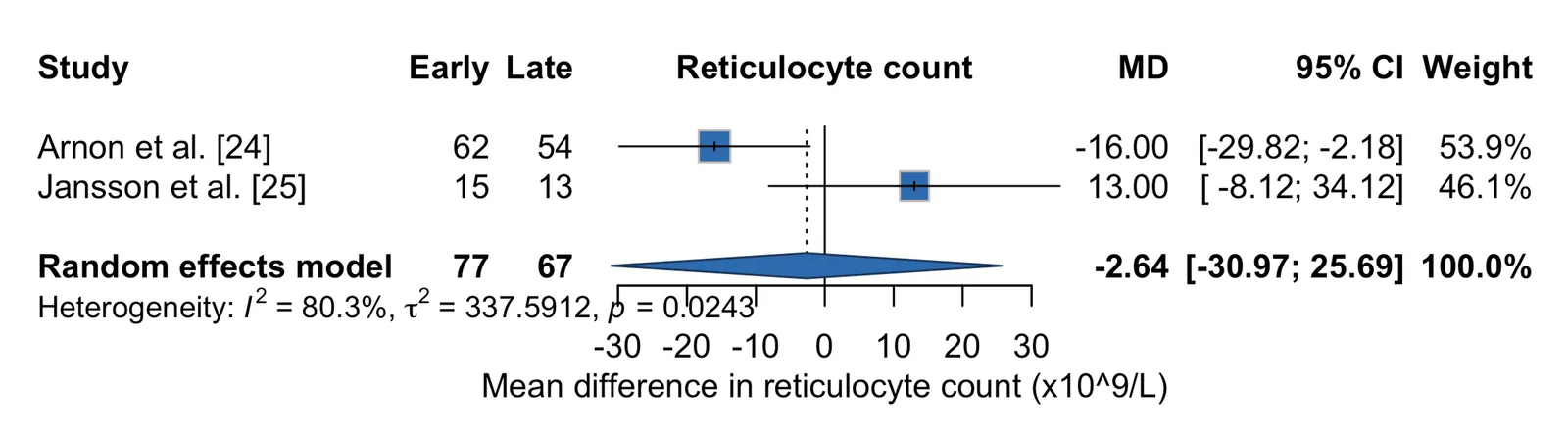
Reticulocyte-count mean difference by timing of iron initiation Forest plot of reticulocyte-count mean difference for early versus late enteral iron initiation. The pooled estimate uses an inverse-variance DerSimonian–Laird random-effects model. Units are ×10⁹/L.

**Table 2:** Pooled estimates for early versus late initiation MD: mean difference; RR: risk ratio; DL: DerSimonian–Laird; HK: Hartung–Knapp; CI: confidence interval.

Outcome	Studies	k	N	Model	Estimate (95% CI)	I²	Heterogeneity Q p
Hemoglobin (MD)	Arnon et al. [24]; Jansson et al. [25]	2	144	DL random	0.85 (0.54, 1.16)	0%	0.47
HK-adjusted	0.85 (-0.60, 2.29)	0%	0.47				
Transfusion (RR)	Arnon et al. [32]; Franz et al. [27]	2	272	DL random	0.38 (0.07, 2.17)	69.5%	0.07
HK-adjusted	0.38 (extremely wide)	69.5%	0.07				
Reticulocytes (MD)	Arnon et al. [24]; Jansson et al. [25]	2	144	DL random	-2.64 (-30.97, 25.69)	80.3%	0.024
HK-adjusted	-2.64 (-186.31, 181.02)	80.3%	0.024				

Planned subgroup analyses were not feasible because fewer than two comparable studies were available within relevant strata. Sensitivity analyses comparing DL and HK models showed that for hemoglobin the HK interval crossed the null, and for transfusion and reticulocytes the HK intervals were extremely wide, confirming that inferences are sensitive to model choice. Formal assessment of publication bias was not performed because each meta-analysis included only two studies, well below the prespecified threshold of ten.

Certainty of Evidence

The GRADE framework [18] was applied to the three pooled outcomes (Table 3). All began at high certainty from randomized trials but were downgraded for risk of bias (one level for all). Inconsistency was not a concern for hemoglobin (I² = 0%) but led to one-level downgrades for transfusion (I² = 69.5%) and reticulocyte count (I² = 80.3%). Imprecision downgraded hemoglobin by one level (HK interval crossed the null) and transfusion and reticulocyte count by two levels (extremely wide intervals). No separate downgrade was applied for publication bias because formal assessment was not possible with only two studies.

**Table 3:** GRADE summary of findings for pooled outcomes MD: mean difference; RR: risk ratio; CI: confidence interval; HK: Hartung–Knapp.

Outcome	Participants (studies)	Effect (95% CI)	Certainty	Main reasons for downgrading
Hemoglobin (MD)	144 (2)	0.85 (0.54, 1.16)	Low	Imprecision (k=2; HK interval crosses null); risk of bias (-1)
Transfusion (RR)	272 (2)	0.38 (0.07, 2.17)	Very low	Imprecision (wide CI, k=2); inconsistency (I²=69.5%); risk of bias (-1)
Reticulocytes (MD)	144 (2)	-2.64 (-30.97, 25.69)	Very low	Imprecision (wide CI, k=2); inconsistency (I²=80.3%); risk of bias (-1)

Discussion

Interpretation in Relation to Guidelines

This review assembled 25 studies evaluating enteral iron strategies in preterm and eligible low-birth-weight infants. The evidence base was dominated by small and clinically heterogeneous trials and cohorts, and most outcomes could not be quantitatively combined because of differences in interventions, outcome definitions, and reporting. Only three outcomes within the early-versus-late initiation comparison were sufficiently comparable for meta-analysis, and each pooled estimate was based on two studies. Earlier initiation yielded higher hemoglobin in the primary random-effects analysis; however, the confidence interval crossed the null under small-sample adjustment. Estimates for blood transfusion and reticulocyte count were imprecise and heterogeneous. Across dose-comparison trials, higher doses were not consistently associated with additional hematological benefit. Taken together, the findings should therefore be regarded as suggestive rather than confirmatory.

These findings are broadly consistent with previous systematic reviews indicating that enteral iron supplementation can improve iron status and reduce iron deficiency or anemia, while effects on growth and neurodevelopment remain less certain and higher doses have not consistently demonstrated additional hematological benefit [4,11]. Contemporary recommendations from WHO and ESPGHAN support enteral iron supplementation in preterm and low-birth-weight infants but differ somewhat in recommended dose, timing, and individualization according to clinical circumstances [9,10].

Biological Rationale and Heterogeneity

A biological rationale for earlier and adequate iron is well grounded: iron is rate-limiting for erythropoiesis and brain development [1,6,7]. Countervailing mechanisms are equally plausible, as supplemental and transfusion-derived iron can increase oxidative stress in preterm infants with immature antioxidant defenses [2,5]. These opposing mechanisms, combined with variation in erythropoietin co-treatment, feeding type, and gestational maturity, plausibly contribute to the observed heterogeneity.

Clinical and Methodological Limitations

Clinical and methodological heterogeneity across the included studies was considerable, reflecting differences in gestational maturity, birth weight, iron formulation, dose, initiation timing, treatment duration, feeding practices, erythropoietin co-intervention, and outcome definitions. Many studies were small, and incomplete or non-standardized outcome reporting prevented quantitative synthesis for most endpoints. Each meta-analysis contained only two studies, resulting in limited precision, unstable estimates of heterogeneity, inability to conduct meaningful subgroup analyses, and inability to formally assess small-study or publication bias. In addition, some evidence retained for narrative synthesis involved prespecified eligible subgroups, complex co-interventions, or non-comparative designs, limiting direct comparability and generalizability. Consequently, the quantitative findings should be considered exploratory and interpreted alongside the low-to-very-low certainty of the underlying evidence.

## Conclusions

Current evidence is insufficient to establish an optimal enteral iron dose or initiation strategy for preterm and VLBW infants. Only three outcomes-hemoglobin, blood-transfusion requirement, and reticulocyte count were sufficiently comparable for quantitative pooling, and each estimate was derived from only two studies. Although earlier initiation was associated with higher hemoglobin in the primary analysis, this finding was not robust to small-sample adjustment, while estimates for transfusion and reticulocyte count were imprecise and heterogeneous. The certainty of evidence was low to very low, precluding firm conclusions regarding an optimal regimen. Clinical decisions should therefore continue to balance the potential benefits of preventing iron deficiency against individual transfusion exposure, iron status, and uncertainty regarding excessive iron exposure. Adequately powered multicenter randomized trials using standardized hematological, neurodevelopmental, and safety outcomes are needed to determine the optimal dose and timing of enteral iron supplementation.
